# Lateral inhibition and odor discrimination in a model of the olfactory bulb

**DOI:** 10.1186/1471-2202-14-S1-P327

**Published:** 2013-07-08

**Authors:** Denise Arruda, Antonio C Roque

**Affiliations:** 1 Departamento de Física, FFCLRP, Universidade de São Paulo, Ribeirão Preto, SP, 14040-901, Brazil

## 

In the olfactory system, odors are detected by olfactory sensory neurons (OSN), which send their axons to glomeruli in the olfactory bulb (OB) where they make excitatory synapses with mitral and periglomerular (PG) cells [1]. The OB has a complex internal circuitry, which displays two levels of lateral inhibition. At the glomerular level, mitral and PG cells interact via dendrodendritic synapses in which mitral cells excite PG cells and PG cells inhibit mitral cells. Deeper at the external plexiform layer, mitral and granule cells also interact via dendrodendritic synapses, with mitral cells exciting granule cells and granule cells inhibiting mitral cells.

Here we present a model of the OB constructed in NEURON [2] to study the effects of PG and granule cells on odor discrimination. The model contains 6 OSNs, 6 mitral cells, 30 PG cells and 240 granule cells. The OSNs are modeled by a 2-compartment adaptation of a previous model [3], the PG cells are modeled by a 6-compartment model recently developed by us [4], and the mitral and granule cell models were taken from [5]. The OB model is organized into 3 glomeruli, each one receiving inputs from a different pair of OSNs. Each glomerulus has 2 mitral cells and 10 PG cells and is associated with 80 granule cells. The cells were connected according to observed patterns of OB synaptic organization, with intra-glomerular synapses between mitral and PG/granule cells and inter-glomerular synapses mediated by PG cells.

The model was stimulated by 5 different odors with different patterns of activation of the 3 glomeruli. Odors 1, 2 and 3 strongly activate distinct glomeruli but odors 4 and 5 activate the 3 glomeruli with overlapping patterns. The model responses to odors were measured by the average firing frequency of the two mitral cells in each glomerulus and the raster plots of the 6 mitral cells. The inhibitory effects of the PG and granule cells on the model response were evaluated by varying the maximum synaptic conductances of their synapses onto mitral cells between 0 and 0.01 microsiemens in steps of 0.001 microsiemens. For each pair of values of these maximum synaptic conductances 10 repetitions of each simulation were done to calculate averages. This allowed us to assess both the independent and collective effects of the PG and granule cells on the responses of the 3 glomeruli to the 5 odors.

Our results show that PG cells alone can efficiently shape the responses of the 3 glomeruli so that they discriminate the 5 odors. With small increases in the maximum conductance of the PG cell inhibitory synapse, the glomeruli that already responded strongly to a single odor had their responses enhanced and the glomeruli that responded to the three odors had their response strength to one of them increased while the responses to the other two decreased. On the other hand, the inhibitory action of the granule cells alone was much less efficient in enhancing the responses of the first three glomeruli to odors 1, 2 and 3 and was not capable of improving the discriminatory capability of the latter two glomeruli to odors 4 and 5. On the contrary, the effect of increasing the maximum conductance of the granule cell synapse was to increase the response overlaps of the latter glomeruli to these odors.
